# Comprehensive predictions of target proteins based on protein-chemical interaction using virtual screening and experimental verifications

**DOI:** 10.1186/1472-6769-12-2

**Published:** 2012-04-05

**Authors:** Hiroki Kobayashi, Hiroko Harada, Masaomi Nakamura, Yushi Futamura, Akihiro Ito, Minoru Yoshida, Shun-ichiro Iemura, Kazuo Shin-ya, Takayuki Doi, Takashi Takahashi, Tohru Natsume, Masaya Imoto, Yasubumi Sakakibara

**Affiliations:** 1 Department of Biosciences and Informatics, Faculty of Science and Technology, Keio University, 3-14-1 Hiyoshi, Kohoku-ku, Yokohama, 223-8522, Japan; 2 Chemical Genetics Laboratory, RIKEN Advanced Science Institute, 2-1 Hirosawa, Wako-shi, Saitama, 351-0198, Japan; 3 National Institute of Advanced Industrial Science and Technology (AIST), 2-4-7 Aomi, Koto-ku, Tokyo, 135-0064, Japan; 4 Graduate School of Pharmaceutical Sciences, Tohoku University, 6-3 Aza-Aoba, Aramaki, Aoba, Sendai, 980-8578, Japan; 5 Department of Applied Chemistry, Tokyo Institute of Technology, 2-12-1 Ookayama, Meguro, Tokyo, 152-8552, Japan

## Abstract

**Background:**

Identification of the target proteins of bioactive compounds is critical for elucidating the mode of action; however, target identification has been difficult in general, mostly due to the low sensitivity of detection using affinity chromatography followed by CBB staining and MS/MS analysis.

**Results:**

We applied our protocol of predicting target proteins combining *in silico* screening and experimental verification for incednine, which inhibits the anti-apoptotic function of Bcl-xL by an unknown mechanism. One hundred eighty-two target protein candidates were computationally predicted to bind to incednine by the statistical prediction method, and the predictions were verified by *in vitro* binding of incednine to seven proteins, whose expression can be confirmed in our cell system.

As a result, 40% accuracy of the computational predictions was achieved successfully, and we newly found 3 incednine-binding proteins.

**Conclusions:**

This study revealed that our proposed protocol of predicting target protein combining *in silico* screening and experimental verification is useful, and provides new insight into a strategy for identifying target proteins of small molecules.

## Background

To understand complex cell systems, functional analysis of proteins has become the main focus of growing research fields of biology in the post-genome era; however, the roles of many proteins in cellular events remain to be elucidated. Among various methods to elucidate protein functions, the approach of chemical genetics is notable, with small molecular compounds used as probes to elucidate protein functions within signal pathways [1,2]. Indeed, several bioactive compounds have led to breakthroughs in understanding the functional roles of proteins [3-11]; however, one significant hurdle to developing new chemical probes of biological systems is identifying the target proteins of bioactive compounds, discovered using cell-based small-molecule screening.

A variety of methods and technologies for identifying target proteins have been reported [12]. Among them, affinity chromatography is often used for identifying biological targets of multiple small molecules of interest; however, it is usually very difficult to identify compound-targeted protein with low expression because of the low sensitivity of detection using coomassie brilliant blue (CBB) staining and MS/MS analysis. Thus, target identification of small molecules using affinity chromatography is severely limited. To overcome the limitations of affinity chromatography, we propose a new protocol combining *in silico* screening and experimental verification for identification of target proteins.

In our previous work, we developed an *in-silico* screening system, called “COPICAT” (Comprehensive Predictor of Interactions between Chemical compounds And Target proteins), to predict the comprehensive interaction between small molecules and target proteins [13]. If a target protein is input in the system, a list of chemical compounds which are likely to interact with the protein is predicted. In our previous work, several potential ligands for the androgen receptor were predicted by this system, these predictions were experimentally verified, and a novel antagonist was found [14]. On the other hand, if a chemical compound is input in the system, a list of proteins which are likely to interact with the compound is predicted by the system.

Previously, we isolated the natural product incednine from the fermentation broth of *Streptomyces* sp. ML694-90F3, which consists of a novel skeletal structure, enol-ether amide in the 24-membered macrolactam core, with two aminosugars. In addition, it was reported that incednine induced apoptosis in Bcl-xL-overexpressing human small cell lung carcinoma Ms-1 cells when combined with several anti-tumor drugs including adriamycin, camptothecin, cisplatin, inostamycin, taxol, and vinblastine [15]. Because this compound inhibits the anti-apoptotic function of Bcl-2/Bcl-xL without affecting its binding to pro-apoptotic Bcl-2 family proteins, it may target other proteins associated with the Bcl-2/Bcl-xL-regulated apoptotic pathway. To address the mode of action of incednine underlying its interesting function, we first synthesized affinity-tagged incednine which is biologically active (data not shown), and proteins bound to incednine were separated by SDS-PAGE followed by CBB staining, and each protein band was directly identified using liquid chromatography-tandem mass (LC-MS/MS) spectrometry analysis. Fifty-three proteins were identified as listed in Table 1, and some of which, such as eukaryotic initiation factor 4A3(eIF4A3), prolyl 4-hydroxylase, beta subunit (PDI), heat shock protein 70 (HSP70), and protein phosphatase 2A (PP2A) were reported to relate to cancer cell survival[16-19]. Therefore these were knocked down by siRNA or inhibited by a specific inhibitor, and assessed for their ability to modulate Bcl-2/Bcl-xL anti-apoptotic function, as does incednine. However, the candidate proteins tested did not appear to be the target responsible for modulating Bcl-2/Bcl-xL anti-apoptotic function (Additional file 1). Therefore, the target protein of incednine responsible for modulating Bcl-2/Bcl-xL anti-apoptotic function has not yet been determined, and further candidate proteins as targets of incednine are expected to emerge.

**Table 1:** List of proteins identified to bind to incednine in our previous binding experiments

**Protein**	**Uniprot ID**	**Kegg ID**
poly 4- hydroxylase, beta submit	P07237	5034
N-acylaminoacyl peptide hydrolase	P13798	327
Heat shock protein 70	P08107	3303/3304
Protein Phosphatase A2	P67775	5515
Similar to DNA damage-binding protein 1	Q16531	1642
Deoxyhypusin synthase isoform alpha	P49366	1725
Methionine adenosyltransferse alpha/beta	P31153/Q00266/Q9NZL9	4144/4143/27430
4-alpha-glucanotransferse	P35573	178
Actin alpha 4	O43707	81
Eukaryotic Initiation factor 4A3	P38919	9775
Deoxycytidine kinase	P27707	1633
ATP synthase H+ transporting, mitochondrial F1complex, alpha	P25705	498
prohibitin	P35232	5245
proteasome alpha 7subuit	O14818	5688
proteasome(prosome,macropain) subunit alpha type 8	Q8TAA3	143471
centaurin,beta 2	Q15057	23527
heterogeneous nuclear ribonucleoprotein A/B	Q99729	3182
heterogeneous nuclear ribonucleoprotein K	P61978	3190
heterogeneous nuclear ribonucleoprotein D	Q14103	3184
heterogeneous nuclear ribonucleoprotein A2/B1	P22626	3181
heterogeneous nuclear ribonucleoprotein A1	P09651	3178
heterogeneous nuclear ribonucleoprotein M	P52272	4670
small nuclear ribonucleoprotein polypeptide D2 family	P62316	6633
mitochondrial riblosomal protein L2	Q5T653	51069
mitochondrial riblosomal protein L20	Q9BYC9	55052
mitochondrial riblosomal protein L3	Q6IBT2	11222
mitochondrial riblosomal protein L40	Q9NQ50	64976
mitochondrial riblosomal protein L46	B2RD75	26589
mitochondrial riblosomal protein L49	B2R4G6	740
mitochondrial riblosomal protein L1	A6NG03	65008
mitochondrial riblosomal protein L37	Q9BZE1	51253
small nuclear ribonucleoprotein-assosiated protein B and B’	P14678	6628
cAMP-dependent protein kinase, regulatory subunit alpha 1	P10644	5573
phosphoribosyl pyrophosphate synthetase-associated protein 1	B2R6M4	5635
peptidylprolyl isomerase-like 2	Q13356	23759
thymoprotein isoform beta, gamma	P42167	7112
fructose-bisphosphate aldolase A	P04075	226
brain creatine kinase	P12277	1152
enolase 1	P06733	2023
Ewing sarcoma breakpoint region 1	Q5THL0	2130
fusion(involved in t(12;16) in malignant liposarcoma)	Q6IBQ5	2521
GDP dissociation inhibitor 2	Q5SX88	2665
nucleosome assembly protein 1-like 1	P55209	4673
nucleosome assembly protein 1-like 4	Q99733	4676
phosphoglycerate dehydrogenase	O43175	26227
triosephosphate isomerase 1	P60174	7167
clathrin heavy chain 1	Q00610	1213
clathrin heavy poly peptide -like 1	P53675	8218
glutamyl-prolyl tRNA synthetase	P07814	2058
retinoblastoma binding protein 7	Q16576	5931
retinoblastoma binding protein 4	Q09028	5928
tripartite motif-containing 28 protein	Q13263	10155
high glucose-regulated protein 8	Q9Y5A9	51441

**Figure 1:**
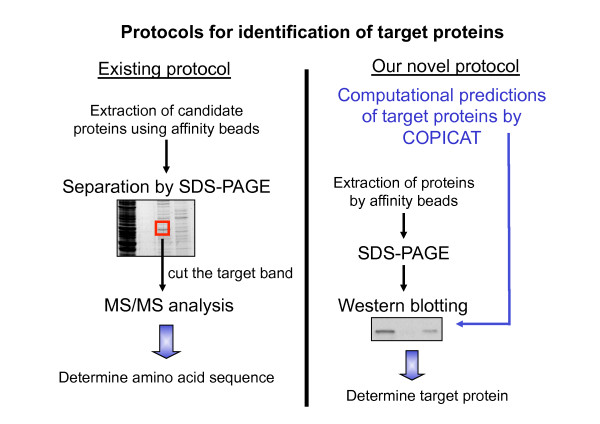
** Schematic illustration of our protocol combining *in silico* screening and experimental verification for identification of target protein.**

In this context, we propose a new protocol combining *in silico* screening and experimental verification for the identification of target proteins. We first predicted the candidate proteins likely binding to the input compound by applying the COPICAT system, and then employed western blotting to detect the binding of predicted proteins to the input compound. This method solves the problem of the low sensitivity of the traditional method (as illustrated in Figure 1).

## Results

### Computational prediction of target proteins for incednine

We set the chemical compound “incednine” as the binding ligand, and candidate proteins for the targets of incednine were computationally predicted from the KEGG database by using the statistical prediction method for protein-chemical interaction. The training dataset of protein-chemical interactions to construct the SVM-based statistical learning model was collected from the approved DrugCards data in the DrugBank database [20], and 53 interactions with incednine obtained from our previous binding experiments using affinity chromatography (see Table 1 and Methods) because the prediction accuracy was increased when more training samples of protein-chemical interactions were given to the SVM-based statistical learning model. Among 24,245 human proteins in the KEGG repository, 182 proteins were newly predicted as positive, that is, to interact with incednine with high probability greater than the 0.5 threshold (the default threshold value).

### Clustering of computationally predicted proteins

The 182 proteins that were computationally predicted to bind to incednine were clustered by the hierarchical clustering method using 199-dimentional feature vector that was used for encoding amino acid sequences to construct the SVM-based statistical learning model (See Methods section for the details). Note that the similarity based on this 199-dimentional feature vector is different from the sequence similarity, and this similarity measure based on the 199-dimentional vector was proven to work well for protein-chemical interaction predictions in our previous work [13]. For example, 5HTT and AR α-1A showed only about 10% sequence similarity although both were reported to interact with the MDMA drug and successfully predicted by our SVM-based statistical learning method. A cutoff threshold on the constructed clustering tree was determined so that the proteins were clustered into 11 clusters and each cluster had a statistically significant number of members. The proteins predicted to bind to incednine are listed in Additional file 2.

### Experimental verification

**Figure 2:**
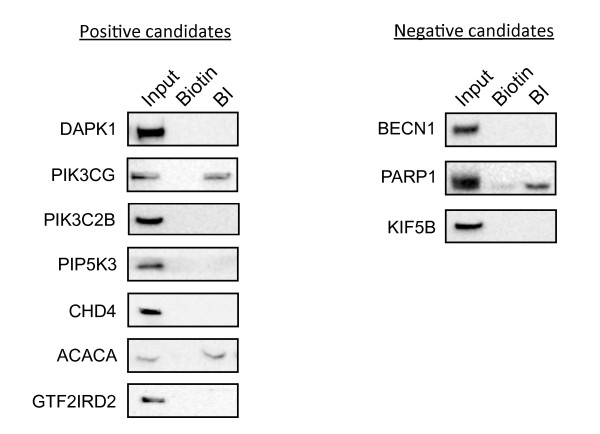
** Experimental verification by *in vitro* biotinylated incednine pull-down assay.** Lysates from Ms-1 overexpressing Bcl-xL were incubated with biotin (Biotin) or biotinylated incednine (BI) and avidin beads for 3 h. The beads were washed, and co-precipitated proteins were eluted with 2 mM biotin. The eluted proteins were subjected to western blotting using the indicated antibodies. “Positive candidates” means proteins which were predicted to bind to incednine, and “Negative candidates” means proteins which were predicted not to bind to incednine.

Next, to examine whether incednine can bind to the proteins, an *in vitro* biotinylated incednine pull-down assay using the lysate of Bcl-xL expressing Ms-1 cells was performed. We tested 16 proteins as pilot experiments, which are selected from each cluster by one or two based on antibody availability. Negative candidates that were predicted not to bind to incednine were extracted for experimental verification. These proteins, positive candidates and negative candidates, are listed in Table 2. Among positive candidate proteins, 2 positive candidates PIK3CG and ACACA were found to bind to incednine, and 5 positive candidates DAPK1, PIK3C2B, PIP5K3, CHD4, GTF2IRD2 did not bind to incednine. Among negative candidate proteins, 2 negative candidates BECN1 and KIF5B did not bind to incednine, and 1 negative candidate PARP1 did bind to incednine (Figure 2). On the other hand, ITPR1, PARP14, PLCB1, KIF1A, KIF21B, and RGPD5, listed as positive candidates in Table 2, were not well expressed and were not detected in Bcl-xL-expressing Ms-1 cells; therefore, accuracy of 40% (4/10), sensitivity of 66.7% (2/3) and precision of 28.6% (2/7) were achieved.

**Table 2:** Representative proteins selected from each cluster and negative candidates for experimental verification

**Cluster No.**	**Representative Protein**
1	ITPR1 (inositol 1,4,5-triphosphate receptor, type 1)
2	DAPK1 (death-associated protein kinase 1)
3	PIK3CG (phosphoinositide-3-kinase, catalytic, gamma polypeptide), PIK3C2B (phosphoinositide-3-kinase, class 2, beta polypeptide)
4	PARP14 (poly (ADP-ribose) polymerase family, member 14)
5	PIP5K3 (phosphatidylinositol-3-phosphate/phosphatidylinositol 5-kinase, type III)
6	PLCB1 (phospholipase C, beta 1)
7	CHD4 (chromodomain helicase DNA binding protein 4)
8	KIF1A (kinesin family member 1A), KIF21B (kinesin family member 21B)
9	ACACA (acetyl-Coenzyme A carboxylase alpha)
10	GTF2IRD2 (GTF2I repeat domain containing 2)
11	RGPD5 (RANBP2-like and GRIP domain-containing protein 5)
Negative	Proteins predicted not to bind to incednine
1	BECN1 (Beclin-1)
2	PARP1 (poly (ADP-ribose) polymerase family, member 1)
3	KIF5B (kinesin family member 5B)

## Discussion

For target identification using affinity chromatography, conventional method requires multiple steps as follows; SDS-PAGE, CBB staining, excision of gel, destaining, reduction, trypsinization, and application to LC-MS/MS system (7 steps); these steps can be cumbersome, time-consuming and require expensive installation. Furthermore, CBB staining used in conventional method can detect proteins over nanogram order. In contrast, our proposed protocol for predicting target protein allows us to use western blotting to detect proteins in picogram order. Indeed, we found two incednine-binding proteins by this prediction. Additionally, we can enhance the precision of COPICAT by feeding back the experimental results to the system.

In this work, PIK3CG, PARP1, and ACACA were revealed to bind to incednine by applying our protocol to identify potential target proteins of chemical compounds. These proteins are potential targets of incednine because it has been reported that these proteins are related to cancer survival and drug resistance, as follows.

PI3KCG encodes p110 catalytic subunit isoform p110γ and heterodimerizes with regulatory subunit p101, composing class IB PI3K in the PI3K family [21,22]. Although PIK3CG and PIK3C2B are distant homologous with 20% sequence identity, incednine selectively binds to PIK3CG but not PIK3C2B (Figure 2). In contrast to class IA, class IB PI3K acts downstream of G-protein coupled receptors (GPCR). It has been reported that p110γ was upregulated and activated by the chimeric oncogene Bcr-Abl expression to contribute to cell proliferation and drug resistance in chronic myelogenous leukemia [23], and was found to be highly and specifically expressed among the PI3K family in human pancreatic cancer [24], suggesting that class IB PI3K might relate to cell survival and drug resistance. Product of enzymatic activation of class IB PI3K as class IA, phosphatidylinositol-3,4,5-trisphosphate, makes BAD dissociate from Bcl-xL and promotes cell survival *via* Akt activation [22]. Therefore class IB PI3K might contribute cell survival in Bcl-xL-overexpressing cells.

PARP1 is a member of the PARP protein superfamily that catalyzes the polymerization of ADP-ribose moieties onto target proteins, using NAD^+^ as a substrate and releasing nicotine amide in the process [25]. PARP1 activity is important for the regulation of homeostasis and the maintenance of genomic stability, participating in DNA repair, the regulation of transcription, DNA replication, cell differentiation, proliferation and cell death [26-28]. Many *in vitro* and *in vivo* experiments demonstrated that inhibition of PARP1 potentiates the cytotoxicity of anti-cancer drugs and ionizing radiation [29-32]. Therefore, incednine could bind to PARP1 and could function as antagonist of anti-apoptotic PARP1 protein. Alternatively, PARP1 is emerging as an important activator of caspase-independent cell death. It has been previously reported that PARP1 mediates the release of apoptosis-inducing factor (AIF), one of the initiators of caspase-independent cell death, possibly due to enzymatic over-activation [33-35]. We also observed that co-treatment of Bcl-xL-overexpressing Ms-1 cells with incednine and ant-tumor drugs induced AIF release and subsequent caspase-independent cell death (unpublished data); therefore, we can not exclude the possibility that incednine binds to PARP1 and functions as PARP1 agonist by accerelating AIF release.

However, the most likely candidate of an incednine target protein is ACACA (acetyl-CoA carboxylase-α), which was classified in cluster 9. ACACA is the rate-limiting enzyme for long-chain fatty acid synthesis that catalyzes the ATP-dependent carboxylation of acetyl-CoA to malonyl-CoA, playing a critical role in cellular energy storage and lipid synthesis [36]. There is strong evidence that cancer cell proliferation and survival are dependent on *de novo* fatty acid synthesis [37-40]. Additionally, ACACA is upregulated in multiple types of human cancers [41,42]; therefore, ACACA may also contribute to cell survival in Bcl-xL-overexpressing tumor cells. Indeed, our preliminary experiments suggested that chemical inhibition of ACACA using TOFA (5-tetradecyloxy-2-furoic acid, ACACA antagonist) or small interfering RNA-mediated ACACA silencing results in the induction of apoptosis in Bcl-xL-overexpressing human small cell lung carcinoma Ms-1 cells when combined with anti-tumor drugs as does incednine (unpublished observation), suggesting that ACACA might be a molecular target of incednine. The possibility that incednine targets ACACA is being actively investigated.

While our experimental verification implied the relatively low precision value 28.6% (2/7), new detections of two incednine-binding proteins in addition to previously identified 53 proteins are significant. On the other hand, while we selected 7 candidates by clustering 182 predicted proteins for experimental verification, more comprehensive verification experiments for the 182 predicted proteins are needed.

The application of our method to incednine resulted in 28.6% (2/7) precision according to *in vitro* pull-down assay. However, this relatively low precision value does not represent the true statistical significance of the method and is not comparable to the benchmark performances (including 98.4% precision) by 10-fold cross-validation for COPICAT system.

This 28.6% precision can be evaluated by using the following *P*-value.

(1) $$P-value=\sum_{x=p}^{t}\frac{{}_{M}C_{x}\times_{\left(N-M\right)}C_{\left(t-x\right)}}{{}_{N}C_{t}}$$

Here, *N* is the number of human proteins, *M* is the number of proteins potentially binding to the incednine, *t* is the number of tested proteins, and *p* is the number of true positives. With *N* =24,245, which is the number of human proteins in the KEGG repository, and *M* = *N* × 1%≒243, which is based on the overestimated assumption that 1% of all proteins could be regarded as potential binding proteins for the incednine. This *P*-value defines the probability that the prediction precision can be obtained by random selection of proteins. Then, *P*-value of 0.002 was obtained for the prediction precision 28.6%. This small *P*-value means that 28.6% (2/7) precision can be obtained with very small chance by random selection, and therefore, this small *P*-value proves the validity of our method.

## Conclusions

Although further study is required for complete determination of the target protein of incednine, this study demonstrated that our proposed protocol of predicting target protein combining *in silico* screening and experimental verification is useful, and provides new insight into a strategy for identifying target proteins of small molecules.

## Methods

### Training datasets

The DrugBank dataset was constructed from Approved DrugCards data, which were downloaded from the DrugBank database [20]. These data consist of 964 approved drugs and their 456 associated target proteins, constituting 1,731 interacting pairs or positives. Additional data about 53 interactions with incednine, listed in Table 1, were obtained from our previous binding experiments.

### Feature vectors

An amino acid sequence of protein is divided into trimers (three amino acid residues), and all of the 8,000 trimers are clustered into 199 groups according to physical-chemical properties. Then, an amino acid sequence is converted to a 199-dimensional feature vector based on the frequencies of 199 clusters (See for [13] the details of this procedure). A chemical compound is also converted to another feature vector of 199 dimension representing substructure statistics extracted from the structural formula of a chemical compound. The size of the dimensions, that is, 199 dimensions, was determined based on the variance of each dimension. The top 199 dimensions with significantly diverse variances in statistical classification were selected.

### Statistical prediction method for protein-chemical interaction

We developed a comprehensively applicable statistical prediction method for interactions between any proteins and chemical compounds, which requires only protein sequence data and chemical structure data and utilizes the statistical learning method of Support Vector Machines (SVM)[13,14].

We consider the problem as the binary classification of protein-chemical pairs whose abstractive identities are represented numerically by the 199 dimensional feature vectors defined above. We obtained a “positive” sample set, i.e., a set of protein-chemical pairs that have been proven to interact with each other via biological assays, from the DrugBank database [20]. Along with the positive sample set, SVM-based classifiers require a “negative” sample set, i.e., a set of protein-chemical pairs that do not interact with each other. Such a negative sample set can be extracted randomly from the whole complement set of the positive sample set. Though we used random pairs of drugs and proteins as negative samples in constructing a model, the lack of reliable negative samples is always a problem when applying the statistical learning methods. In our current study, it is assumed that drugs in the DrugBank dataset rarely interact with proteins other than their known targets because they are approved drugs. Using the resultant positive and negative protein-chemical pair sets, we trained two-layer SVMs. First, we trained each multiple first-layer SVM with small sample sets designed with different criteria. Next, using another larger sample set, we trained a second-layer SVM whose input is a set of probabilities output from the firstlayer SVMs. The prediction performances were evaluated by 10-fold cross-validation using the DrugBank dataset. The sensitivity, specificity, precision, and accuracy were 0.954, 0.999, 0.984, and 0.997, respectively, in cross-validation. The details of the algorithms and their prediction accuracy are described in our previous reports [13,14].

### Support vector machines

Given *n* samples, each of which has an *m*-dimensional feature vector $\left(x_{i}=\left(x_{i}^{1},\ldots ,x_{i}^{m}\right)\right)$ and one of two classes, such as binding and non-binding $\left(y\in \{1,-1\}\right)$, an SVM produces the classifier

(2) $$f(x)=sign\left(\sum_{i=1}^{n}\alpha_{i}y_{i}K\left(x_{i},x\right)+b\right)\text{,}$$

where *x* is any new object which needs to be classified, *K* (·,·) is a kernel function which indicates that the similarity between two vectors and (α_1_,⋯,α_n_) are the learned parameters. The RBF kernel $K\left(S_{1},S_{2}\right)=\exp\left(-\gamma ∥S_{1}-S_{2}∥\right)$ was utilized for the SVM classifier. In our study, the LIBSVM program [43] was employed to construct the SVM model.

### Cell culture

Bcl-xL-overexpressing human SCLC Ms-1 cells [15] were maintained in Rosewell Park Memorial Institute media (Nissui, Japan) supplemented with 5% fetal bovine serum, 100 U/ml penicillin G, and 0.1 mg/mL kanamycin at 37°C in a humidified 5% CO_2_ atmosphere.

### Antibodies

Mouse monoclonal anti-DAPK1 (DAPK-55), rabbit monoclonal anti-PIK3CG (Y388), rabbit monoclonal anti-ACACA (EP687Y), mouse monoclonal anti-PIK3C2B, rabbit polyclonal anti-ITPR1, mouse monoclonal anti-PIP5K3, mouse monoclonal anti-CHD4, mouse polyclonal anti-GTF2IRD2, mouse polyclonal anti-PLCB1 antibodies were purchased from Abcam (Cambridge, MA). Rabbit polyclonal anti-KIF21B and mouse monoclonal anti-KIF5B (clone H2) antibodies were purchased from Millipore (Bedford, MA). Goat polyclonal anti-PARP14 and goat polyclonal anti-KIF1A were purchased from Santa Cruz Biotechnology (Santa Cruz, CA). Mouse monoclonal anti-Beclin (clone 20) antibody was purchased from BD Transduction Laboratories (San Diego, CA). Rabbit polyclonal anti-PARP1 antibody was purchased from Cell Signaling Technology (Beverly, MA). Rabbit polyclonal anti-RGPD5 antibody was purchased from Lifespan Biosciences (Seattle, WA). Mouse monoclonal anti-Flag (M2) antibody was purchased form Sigma (St. Louis, MO).

Horseradish peroxidase-conjugated anti-mouse IgG and anti-rabbit IgG secondary antibodies were purchased from GE Healthcare (Little Chalfont, UK). Horseradish peroxidase-conjugated anti-goat IgG was purchased from Santa Cruz Biotechnology.

### Western blotting

Cell lysates were separated by SDS-PAGE and transferred to a PVDF membrane (Millipore) by electroblotting. After the membranes had been incubated with primary and secondary antibodies, the immune complexes were detected with an Immobilon Western kit (Millipore), and luminescence was detected with a LAS-1000 mini (Fujifilm, Tokyo, Japan).

### Preparation of incednine and biotinylated incednine

Incednine was isolated from the culture broth of *Streptomyces* sp. ML694-90F3 [15]. To obtain biotinylated incednine (see Additional file 3), incednine (137.0 mg) and the amine-reactive biotin-X (100.0 mg; Invitrogen) were dissolved in 13.0 mL CHCl_3_:MeOH (10:1). After stirring at 40°C for 20 h, the reaction mixture was concentrated to dryness. The residue was resolved in 50 mL CHCl_3_:MeOH:H_2_O (5:6:4) and partitioned three times under basic conditions. The lower layer of CHCl_3_:MeOH:H_2_O (5:6:4) was evaporated *in vacuo* to yield a brown residue. The residue was purified by HPLC (Senshu Pak Pegasil ODS 30 x 250 mm) and eluted with MeOH:40 mM KH_2_PO_4_ aq. (70:30) to give 19.4 mg biotinylated incednine.

### *In vitro* biotinylated incednine pull-down assay

Bcl-xL-overexpressiong Ms-1 cells were collected and sonicated twice in IP buffer (50 mM HEPES (pH 7.5), 150 mM NaCl, 2.5 mM EGTA, 1 mM EDTA, 1 mM DTT, and a protease inhibitor cocktail (Roche, Mannheim, Germany)) for 10 s. The cell lysates were centrifuged at 10,000 *g* for 15 min at 4°C. The resulting supernatants were incubated with biotin (50 nmol) or biotinylated incednine (50 nmol) and avidin beads at 4°C for 3 h. The beads were washed three times with phosphate-buffered saline (PBS). The bound proteins were eluted with 2 mM biotin in PBS, and concentrated by a centrifugal filter device (Ultracel (YM-10); Millipore). The resulting proteins were boiled in SDS sample buffer for 5 min and subjected to western blotting.

### Liquid chromatography-tandem mass spectrometry

Incednine binding proteins purified using biotinylated incednine / avidin beads, and flag-tagged incednine (see Additional file 4) / anti-Flag antibody were anaylzed by liquid chromatography-tandem mass spectrometry (LC–MS/MS) system as previously described, respectively [44,45].

## Authors’ contributions

YS and MI designed the study and analyzed the data. HK, HH, MN and YF performed the experiments. YS, MI and HK wrote the paper. YF synthesized biotinylated incednine. AI, MY, SI, KS, TD, TT, and TN performed MS/MS analysis. All authors read and approved the final manuscript.

## Supplementary Material

Additional file 1 Validation work for eIF4A3, PDI, PP2A and Hsp70. Click here for file

Additional file 2 Proteins computationally predicted to bind to incednine (grouped into 11 clusters). Click here for file

Additional file 3 A stucture of biotinylated incednine. Click here for file

Additional file 4 ** Preparation of Flag-tagged Incednine**[46,47]. Click here for file
