# Supplementary material for: Comprehensive predictions of target proteins based on protein-chemical interaction using virtual screening and experimental verifications
Source: BMC Chem Biol. 2012 Apr 5;12:2. doi: 10.1186/1472-6769-12-2 (PMC3471015; doi:10.1186/1472-6769-12-2)
Supplement: Additional file 1 — Validation work for eIF4A3, PDI, PP2A and Hsp70. [file 1472-6769-12-2-S1.pdf]

Additional file 1 Validation work for eIF4A3, PDI, Hsp70, and PP2A

Supplementary Figure Legends

Figure S1. Knockdown of eIF4A3 did not affect cell viability in Bcl-xL-overexpressing-Ms-1 cells treated with inostamycin. Bcl-xL-overexpressing-Ms-1 cells were transfected with control or eIF4A3 siRNA, and were cultured for 60 h. Then the cells were subjected to western blotting using indicated antibodies (A) or the cells were treated with 0.3 μg/mL inostamycin (INM) for 24 h, and cell viability was assessed by trypan blue dye exclusion assay (B). Values are means of three samples: bars, SD.

Figure S2. Knockdown of PDI did not affect cell viability in Bcl-xL-overexpressing-Ms-1 cells treated with inostamycin. Bcl-xL-overexpressing-Ms-1 cells were transfected with control or PDI siRNA, and were cultured for 60 h. Then the cells were subjected to western blotting using indicated antibodies (A) or the cells were treated with 0.3 μg/mL inostamycin (INM) for 48 h, and cell viability was assessed by trypan blue dye exclusion assay (B). The cell viability of control siRNA-treated cells exposured to 0.3 μg/mL of inostamycin and 80 ng/mL incednine (ICN) was assessed as positive control. Values are means of three samples: bars, SD.

Figure S3. Inhibition of Hsp70 and Inhibition of PP2A did not affect cell viability in Bcl-xL-overexpressing-Ms-1 cells treated with inostamycin. Bcl-xL-overexpressing-Ms-1 cells were treated with Hsp70 inhibitor, KNK437 (A) or PP2A inhibitor, okadaic acid (B), in the absence or presence of 0.3 μg/mL inostamycin (INM). After incubation for 48 h, cell viability was assessed by trypan blue dye exclusion assay (incednine alone; open circle, combination; closed circle). Values are means of three samples: bars, SD.

Figure S1

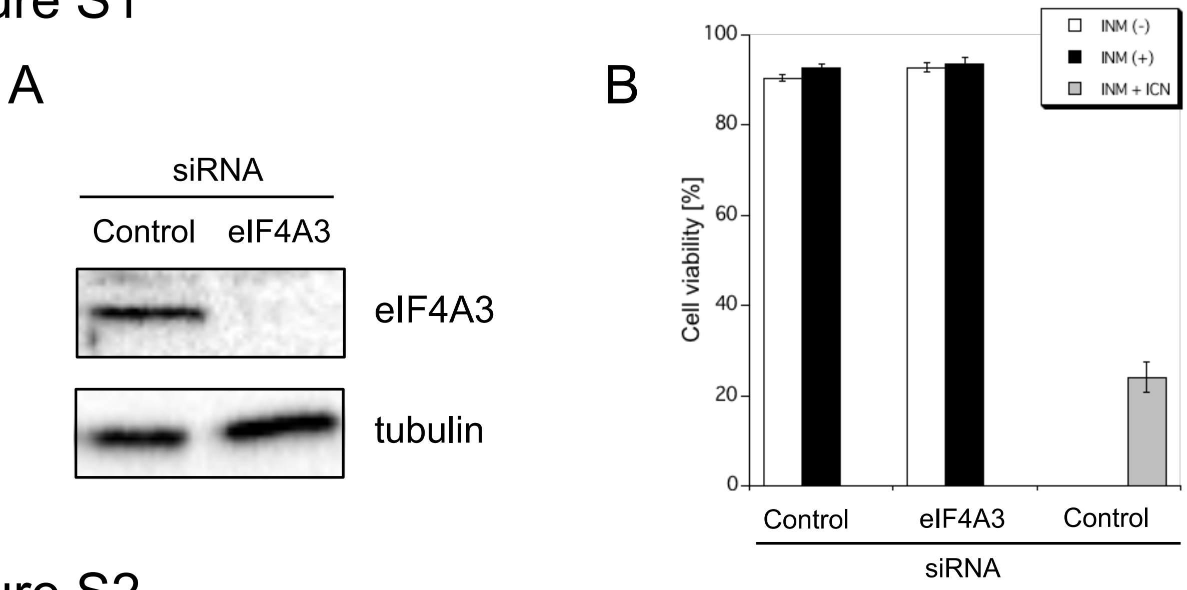

Figure S2

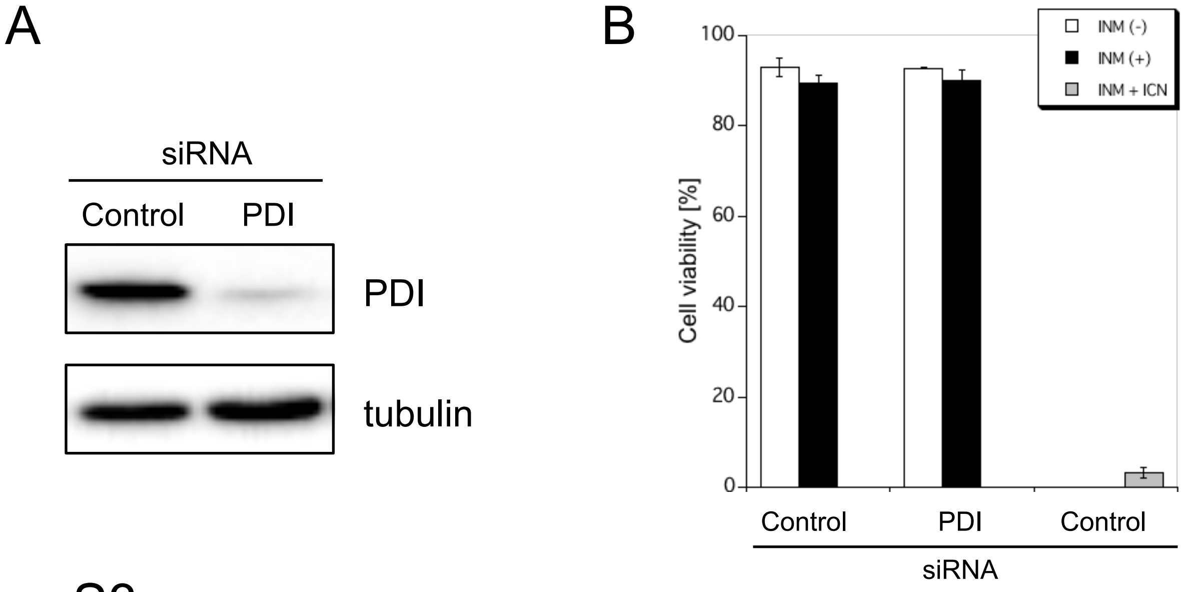

Figure S3

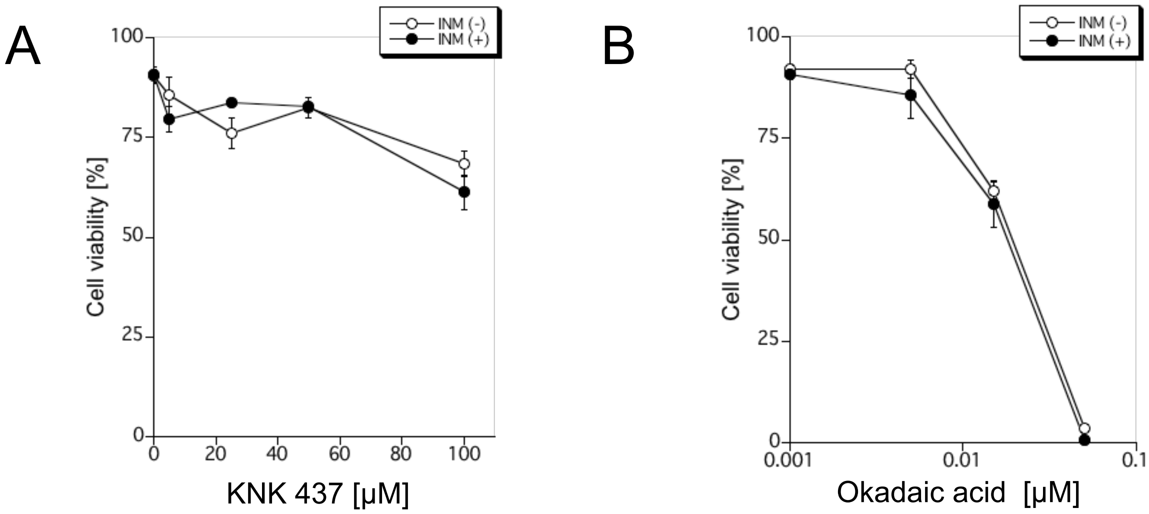

Supplementary Methods

Materials

Okadaic acid was purchased from Sigma (St. Louis, MO). KNK437 was purchased from Calbiochem (Darmstadt, Germany). Rabbit polyclonal anti-eIF4A3 antibody was purchased from Abcam (Cambridge, MA). Mouse monoclonal anti-PDI antibody was purchased from BD Biosciences (Franklin Lakes, NJ)

RNA interference

siRNA for control (12935-300), PDI (HSS143215), and eIF4A3 (HSS114711) were purchased from Invitrogen (Carlsbad, CA). Bcl-xL-overexpressing Ms-1 cells were transfected with 25 nM of siRNA using Lipofectamine 2000 (Invitrogen) according to the manufacturer's instructions.

Trypan blue dye exclusion assay

Collected cells were stained by trypan blue dye (Sigma) in 1.5 mL tube. The cells were counted using a dual-chamber hemocytometer and a light microscope. Total cells and dead cells (stained) were recorded separately, and the percentage of cell viability was calculated as follows:

Cell viability (%) = (total cells – dead cells) / total cells x 100
