# Supplementary material for: Comprehensive predictions of target proteins based on protein-chemical interaction using virtual screening and experimental verifications
Source: BMC Chem Biol. 2012 Apr 5;12:2. doi: 10.1186/1472-6769-12-2 (PMC3471015; doi:10.1186/1472-6769-12-2)
Supplement: Additional file 2 — Proteins computationally predicted to bind to incednine (grouped into 11 clusters). [file 1472-6769-12-2-S2.pdf]

Additional file 2 Proteins computationally predicted to bind to incednine (grouped into 11 clusters)

List of proteins predicted to bind to incednine (Cluster 1)

keggID	protein name
hsa:55127	HEATR1; HEAT repeat containing 1
hsa:9735	KNTC1; kinetochore associated 1
hsa:154664	ABCA13; ATP-binding cassette, sub-family A (ABC1), member 13 ; K05647 ATP binding cassette, subfamily A (ABC1), member 13
hsa:23165	NUP205, C7orf14; nucleoporin 205kDa
hsa:133558	FLJ40243; hypothetical protein FLJ40243
hsa:3071	NCKAP1L, HEM1; NCK-associated protein 1-like ; K05750 NCK-associated protein 1
hsa:23198	PSME4; proteasome (prosome, macropain) activator subunit 4 ; K06699 proteasome activator subunit 4
hsa:10128	LRPPRC, LSFC; leucine-rich PPR-motif containing
hsa:651921	LOC651921; similar to ataxia telangiectasia and Rad3 related protein
hsa:23019	CNOT1, NOT1; CCR4-NOT transcription complex, subunit 1
hsa:120892	LRRK2, PARK8; leucine-rich repeat kinase 2 ; K08844 leucine-rich repeat kinase 2 [EC:2.7.11.1]
hsa:2475	FRAP1, FRAP, FRAP2; FK506 binding protein 12-rapamycin associated protein 1 ; K07203 FKBP12-rapamycin complex-associated protein
hsa:80208	SPG11, KIAA1840; spastic paraplegia 11 (autosomal recessive)
hsa:374467	C12orf63; chromosome 12 open reading frame 63
hsa:8295	TRRAP; transformation/transcription domain-associated protein ; K08874 transformation/transcription domain-associated protein
hsa:23317	DNAJC13; DnaJ (Hsp40) homolog, subfamily C, member 13 ; K09533 DnaJ homolog, subfamily C, member 13
hsa:545	ATR; ataxia telangiectasia and Rad3 related ; K06640 ataxia telangiectasia and Rad3 related [EC:2.7.11.1]
hsa:3710	ITPR3; inositol 1,4,5-triphosphate receptor, type 3 ; K04960 inositol 1,4,5-triphosphate receptor, type 3
hsa:3709	ITPR2; inositol 1,4,5-triphosphate receptor, type 2 ; K04959 inositol 1,4,5-triphosphate receptor, type 2
hsa:3708	ITPR1, SCA15, SCA16; inositol 1,4,5-triphosphate receptor, type 1 ; K04958 inositol 1,4,5-triphosphate receptor, type 1
hsa:4648	MYO7B; myosin VIIB ; K10359 myosin VII

List of proteins predicted to bind to incednine (Cluster 2)

keggID	protein name
hsa:9980	DOPEY2, C21orf5; dopey family member 2
hsa:3064	HTT, HD; huntingtin ; K04533 huntingtin
hsa:2194	FASN; fatty acid synthase (EC:2.3.1.85); K00059 3-oxoacyl-[acyl-carrier protein] reductase [EC:1.1.1.100]; K00209 enoyl-[acyl-carrier-protein] reductase (NADPH2, B-specific) [EC:1.3.1.10]; K00644 [acyl-carrier-protein] S-acetyltransferase [EC:2.3.1.38]; K00645 [acyl-carrier-protein] S-malonyltransferase [EC:2.3.1.39]; K00646 3-oxoacyl-[acyl-carrier-protein] synthase [EC:2.3.1.41]; K00665 fatty-acid synthase [EC:2.3.1.85]; K01071 oleoyl-[acyl-carrier-protein] hydrolase [EC:3.1.2.14]; K01717 3-hydroxypalmitoyl-[acyl-carrier-protein] dehydratase [EC:4.2.1.61]
hsa:168507	PKD1L1; polycystic kidney disease 1 like 1 ; K04987 polycystin 1L
hsa:23523	CABIN1; calcineurin binding protein 1
hsa:23113	PARC; p53-associated parkin-like cytoplasmic protein
hsa:25962	KIAA1429; KIAA1429
hsa:8729	GBF1; golgi-specific brefeldin A resistance factor 1
hsa:23392	KIAA0368; KIAA0368
hsa:57448	BIRC6; baculoviral IAP repeat-containing 6 ; K10586 baculoviral IAP repeat-containing protein 6 (apollon) [EC:6.3.2.19]
hsa:790	CAD; carbamoyl-phosphate synthetase 2, aspartate transcarbamylase, and dihydroorotase (EC:2.1.3.2 3.5.2.3 6.3.5.5); K00609 aspartate carbamoyltransferase catalytic chain [EC:2.1.3.2]; K01465 dihydroorotase [EC:3.5.2.3]; K01955 carbamoyl-phosphate synthase large chain [EC:6.3.5.5]; K01956 carbamoyl-phosphate synthase small chain [EC:6.3.5.5]
hsa:23274	CLEC16A, KIAA0350; C-type lectin domain family 16, member A
hsa:23268	DNMBP; dynamin binding protein
hsa:26005	C2CD3; C2 calcium-dependent domain containing 3
hsa:721	C4B; complement component 4B (Childo blood group) ; K03989 complement component 4
hsa:57705	WDFY4, C10orf64; WDFY family member 4
hsa:720	C4A; complement component 4A (Rodgers blood group) ; K03989 complement component 4
hsa:22878	KIAA1012; KIAA1012
hsa:5189	PEX1; peroxisomal biogenesis factor 1
hsa:4763	NF1; neurofibromin 1 ; K08052 neurofibromin 1
hsa:9044	BTAF1; BTAF1 RNA polymerase II, B-TFIID transcription factor-associated, 170kDa (Mot1 homolog, S. cerevisiae)
hsa:10129	FRY, C13orf14; furry homolog (Drosophila
hsa:8924	HERC2; hect domain and RLD 2 ; K10595 E3 ubiquitin-protein ligase HERC2 [EC:6.3.2.19
hsa:23033	DOPEY1, KIAA1117; dopey family member 1
hsa:253959	GARNL1; GTPase activating Rap/RanGAP domain-like
hsa:1795	DOCK3; dedicator of cytokinesis 3 ; K05727 dedicator of cytokinesis 1/2/3/4/5
hsa:25831	HECTD1; HECT domain containing
hsa:8925	HERC1; hect (homologous to the E6-AP (UBE3A) carboxyl terminus) domain and RCC1 (CHC1)-like domain (RLD) 1 ; K10594 E3 ubiquitin-protein ligase HERC1 [EC:6.3.2.19]

List of proteins predicted to bind to incednine (Cluster 2) (continued)

keggID	protein name
hsa:285527	FRYL, KIAA0826; FRY-like
hsa:23049	SMG1; PI-3-kinase-related kinase SMG-1 ; K08873 PI-3-kinase-related kinase SMG-1
hsa:5286	PIK3C2A; phosphoinositide-3-kinase, class 2, alpha polypeptide (EC:2.7.1.137); K00923 phosphatidylinositol-4-phosphate 3-kinase [EC:2.7.1.154]
hsa:57578	KIAA1409; KIAA1409
hsa:1612	DAPK1; death-associated protein kinase 1 (EC:2.7.1.-); K08803 death-associated protein kinase [EC:2.7.11.1]
hsa:1657	DMXL1; Dmx-like 1
hsa:8518	IKBKAP, DYS; inhibitor of kappa light polypeptide gene enhancer in B-cells, kinase complex-associated protein
hsa:9732	DOCK4; dedicator of cytokinesis 4 ; K05727 dedicator of cytokinesis 1/2/3/4/5
hsa:57186	C20orf74; chromosome 20 open reading frame 74
hsa:57221	KIAA1244, C6orf92; KIAA1244
hsa:23358	USP24; ubiquitin specific peptidase 24
hsa:81846	SBF2, CMT4B2; SET binding factor
hsa:440275	EIF2AK4; eukaryotic translation initiation factor 2 alpha kinase 4 ; K08860 eukaryotic translation initiation factor 2-alpha kinase [EC:2.7.11.1]
hsa:79705	LRRK1; leucine-rich repeat kinase 1 ; K08843 leucine-rich repeat kinase 1 [EC:2.7.11.1]
hsa:57674	RNF213, C17orf27; ring finger protein 21
hsa:283450	C12orf51; chromosome 12 open reading frame 51 (EC:6.3.2.-
hsa:987	LRBA; LPS-responsive vesicle trafficking, beach and anchor containing

List of proteins predicted to bind to incednine (Cluster 3)

keggID	protein name
hsa:778	CACNA1F, CSNB2; calcium channel, voltage-dependent, L type, alpha 1F subunit ; K04853 calcium channel, voltage-dependent, L type, alpha 1F subunit
hsa:21	ABCA3, ABC3; ATP-binding cassette, sub-family A (ABC1), member 3 ; K05643 ATP-binding cassette, subfamily A (ABC1), member 3
hsa:10351	ABCA8; ATP-binding cassette, sub-family A (ABC1), member 8 ; K05650 ATP-binding cassette, subfamily A (ABC1), member 8
hsa:776	CACNA1D, CCHL1A2, CACNL1A2; calcium channel, voltage-dependent, L type, alpha 1D subunit ; K04851 calcium channel, voltage-dependent, L type, alpha 1D subuni
hsa:26154	ABCA12; ATP-binding cassette, sub-family A (ABC1), member 12 ; K05646 ATP-binding cassette, subfamily A (ABC1), member 12
hsa:7223	TRPC4; transient receptor potential cation channel, subfamily C, member 4 ; K04967 transient receptor potential cation channel, subfamily C, member
hsa:50506	DUOX2; dual oxidase 2
hsa:777	CACNA1E, CACNL1A6; calcium channel, voltage-dependent, R type, alpha 1E subunit ; K04852 calcium channel, voltage-dependent, R type, alpha 1E subunit
hsa:775	CACNA1C, CCHL1A1, CACNL1A1; calcium channel, voltage-dependent, L type, alpha 1C subunit ; K04850 calcium channel, voltage-dependent, L type, alpha 1C subunit
hsa:6326	SCN2A, SCN2A1, SCN2A2; sodium channel, voltage-gated, type II, alpha subunit ; K04834 sodium channel, voltage-gated, type II, alpha
hsa:10396	ATP8A1; ATPase, aminophospholipid transporter (APLT), class I, type 8A, member 1 (EC:3.6.3.1); K01530 phospholipid-translocating ATPase [EC:3.6.3.1]
hsa:9716	AQR; aquarius homolog (mouse)
hsa:6098	ROS1; c-ros oncogene 1, receptor tyrosine kinase (EC:2.7.10.1); K05088 proto-oncogene tyrosine-protein kinase ROS [EC:2.7.10.1
hsa:24	ABCA4, STGD1, ABCR, RP19, STGD; ATP-binding cassette, sub-family A (ABC1), member 4 ; K05644 ATP-binding cassette, subfamily A (ABC1), member 4
hsa:140469	MYO3B; myosin IIIB ; K08834 myosin III [EC:2.7.11.1]
hsa:5294	PIK3CG; phosphoinositide-3-kinase, catalytic, gamma polypeptide (EC:2.7.1.137); K00922 phosphatidylinositol-4,5-bisphosphate 3-kinase [EC:2.7.1.153]
hsa:5287	PIK3C2B; phosphoinositide-3-kinase, class 2, beta polypeptide (EC:2.7.1.137); K00923 phosphatidylinositol-4-phosphate 3-kinase [EC:2.7.1.154]
hsa:20	ABCA2, ABC2; ATP-binding cassette, sub-family A (ABC1), member 2 ; K05642 ATP-binding cassette, subfamily A (ABC1), member 2
hsa:2324	FLT4; fms-related tyrosine kinase 4 (EC:2.7.10.1); K05097 FMS-like tyrosine kinase 4 [EC:2.7.10.1]
hsa:23654	PLXNB2; plexin B2 ; K06821 plexin B
hsa:7226	TRPM2, TRPC7; transient receptor potential cation channel, subfamily M, member 2 ; K04977 transient receptor potential cation channel, subfamily M, member 2
hsa:19	ABCA1, ABC1, HDLDT1; ATP-binding cassette, sub-family A (ABC1), member 1 ; K05641 ATP-binding cassette, subfamily A (ABC1), member 1

List of proteins predicted to bind to incednine (Cluster 4)

keggID	protein name
hsa:4547	MTTP; microsomal triglyceride transfer protein
hsa:338	APOB; apolipoprotein B (including Ag(x) antigen)
hsa:164045	HFM1, SEC63D1; HFM1, ATP-dependent DNA helicase homolog (S. cerevisiae)
hsa:9793	CKAP5; cytoskeleton associated protein 5
hsa:5923	RASGRF1, GRF1; Ras protein-specific guanine nucleotide-releasing factor 1 ; K04349 Ras guanine nucleotide-releasing factor
hsa:26098	C10orf137; chromosome 10 open reading frame 137
hsa:64800	EFCAB6; EF-hand calcium binding domain
hsa:54625	PARP14; poly (ADP-ribose) polymerase family, member 14
hsa:122402	TDRD9, C14orf75; tudor domain containing 9 (EC:3.6.1.-)
hsa:4649	MYO9A; myosin IXA ; K10360 myosin IX
hsa:2157	F8, F8C; coagulation factor VIII, procoagulant component ; K03899 coagulation factor VIII
hsa:23230	VPS13A, CHAC; vacuolar protein sorting 13 homolog A (S. cerevisiae)
hsa:54464	XRN1; 5'-3' exoribonuclease 1
hsa:90957	DHX57; DEAH (Asp-Glu-Ala-Asp/His) box polypeptide 57
hsa:4308	TRPM1, MLSN1; transient receptor potential cation channel, subfamily M, member 1 ; K04976 transient receptor potential cation channel, subfamily M, member 1
hsa:10565	ARFGEF1; ADP-ribosylation factor guanine nucleotide-exchange factor 1(brefeldin A-inhibited)
hsa:56886	UGCGL1; UDP-glucose ceramide glucosyltransferase-like 1 ; K00754
hsa:4651	MYO10; myosin X
hsa:10564	ARFGEF2; ADP-ribosylation factor guanine nucleotide-exchange factor 2 (brefeldin A-inhibited)
hsa:2909	GRLF1; glucocorticoid receptor DNA binding factor 1 ; K05732 Rho GTPase activating protein 5/ glucocorticoid receptor DNA binding factor 1
hsa:57580	PREX1; phosphatidylinositol 3,4,5-trisphosphate-dependent RAC exchanger 1
hsa:140803	TRPM6, HOMG, HSH; transient receptor potential cation channel, subfamily M, member 6 ; K04981 transient receptor potential cation channel, subfamily M, member 6 [EC:2.7.11.1]
hsa:4217	MAP3K5, MEKK5; mitogen-activated protein kinase kinase kinase 5 ; K04426 mitogen-activated protein kinase kinase kinase 5 [EC:2.7.11.25]
hsa:54832	VPS13C; vacuolar protein sorting 13 homolog C (S. cerevisiae)
hsa:23348	DOCK9; dedicator of cytokinesis 9
hsa:54822	TRPM7; transient receptor potential cation channel, subfamily M, member 7 ; K04982 transient receptor potential cation channel, subfamily M, member 7 [EC:2.7.11.1]

List of proteins predicted to bind to incednine (Cluster 5)

keggID	protein name
hsa:675	BRCA2, FANCD1, FACD, FANCD; breast cancer 2, early onset ; K08775 breast cancer 2 susceptibility protein
hsa:440279	UNC13C; unc-13 homolog C (C. elegans)
hsa:116984	CENTD1; centaurin, delta 1
hsa:84162	KIAA1109; KIAA1109
hsa:5903	RANBP2; RAN binding protein 2
hsa:65250	C5orf42; chromosome 5 open reading frame 42
hsa:200576	PIP5K3; phosphatidylinositol-3-phosphate/phosphatidylinositol 5-kinase, type III (EC:2.7.1.68); K00889 1-phosphatidylinositol-4-phosphate 5-kinase [EC:2.7.1.68]
hsa:51735	RAPGEF6, PDZGEF2; Rap guanine nucleotide exchange factor (GEF) 6 ; K08020 Rap guanine nucleotide exchange factor (GEF) 6
hsa:5783	PTPN13; protein tyrosine phosphatase, non-receptor type 13 (APO-1/CD95 (Fas)-associated phosphatase) (EC:3.1.3.48); K02374 protein tyrosin phosphatase, non-receptor type 13 (PTPN13)
hsa:10721	POLQ; polymerase (DNA directed), theta (EC:2.7.7.7); K02349 DNA polymerase theta subunit [EC:2.7.7.7]
hsa:51196	PLCE1; phospholipase C, epsilon 1 (EC:3.1.4.11); K05860 phospholipase C, epsilon

List of proteins predicted to bind to incednine (Cluster 6)

keggID	protein name
hsa:23224	SYNE2; spectrin repeat containing, nuclear envelope 2
hsa:87	ACTN1; actinin, alpha 1 ; K05699 actinin alpha
hsa:283446	MYO1H; myosin IH ; K10356 myosin I
hsa:55930	MYO5C; myosin VC ; K10357 myosin V
hsa:23236	PLCB1; phospholipase C, beta 1 (phosphoinositide-specific) (EC:3.1.4.11); K05858 phospholipase C, beta [EC:3.1.4.11]
hsa:5336	PLCG2; phospholipase C, gamma 2 (phosphatidylinositol-specific) (EC:3.1.4.11); K05859 phospholipase C, gamma [EC:3.1.4.11]
hsa:4898	NRD1; nardilysin (N-arginine dibasic convertase) (EC:3.4.24.61); K01411 nardilysin [EC:3.4.24.61]
hsa:10735	STAG2; stromal antigen
hsa:23517	SKIV2L2, KIAA0052; superkiller viralicidic activity 2-like 2 (S. cerevisiae) ; K01529
hsa:80217	C10orf79; chromosome 10 open reading frame 79
hsa:1794	DOCK2; dedicator of cytokinesis 2 ; K05727 dedicator of cytokinesis 1/2/3/4/5
hsa:80005	DOCK5; dedicator of cytokinesis 5 ; K05727 dedicator of cytokinesis 1/2/3/4/5
hsa:10973	ASCC3, HELIC1; activating signal cointegrator 1 complex subunit 3 ; K01529
hsa:23020	ASCC3L1; activating signal cointegrator 1 complex subunit 3-like 1 ; K01529

List of proteins predicted to bind to incednine (Cluster 7)

keggID	protein name
hsa:6830	SUPT6H; suppressor of Ty 6 homolog (S. cerevisiae)
hsa:9790	BMS1, BMS1L; BMS1 homolog, ribosome assembly protein (yeast)
hsa:26038	CHD5; chromodomain helicase DNA binding protein 5
hsa:7155	TOP2B; topoisomerase (DNA) II beta 180kDa (EC:5.99.1.3); K03164 DNA topoisomerase II [EC:5.99.1.3]
hsa:1108	CHD4; chromodomain helicase DNA binding protein 4
hsa:7153	TOP2A, TOP2; topoisomerase (DNA) II alpha 170kDa (EC:5.99.1.2 5.99.1.3); K03164 DNA topoisomerase II [EC:5.99.1.3]
hsa:4646	MYO6, DFNA22, DFNB37; myosin VI ; K10358 myosin VI

List of proteins predicted to bind to incednine (Cluster 8)

keggID	protein name
hsa:9928	KIF14; kinesin family member 14 ; K10392 kinesin family member 1/13/14
hsa:9172	MYOM2; myomesin (M-protein) 2, 165kDa
hsa:138474	TAF1L; TAF1 RNA polymerase II, TATA box binding protein (TBP)-associated factor, 210kDa-like ; K03125 transcription initiation factor TFIID subunit D1
hsa:23046	KIF21B; kinesin family member 21B ; K10395 kinesin family member 4/7/21/27
hsa:55023	PHIP, WDR11; pleckstrin homology domain interacting protein
hsa:3910	LAMA4; laminin, alpha 4 ; K06241 laminin, alpha 4
hsa:57680	CHD8, HELSNF1; chromodomain helicase DNA binding protein 8 ; K04494 chromodomain helicase DNA binding protein 8
hsa:84181	CHD6; chromodomain helicase DNA binding protein 6
hsa:8291	DYSF, LGMD2B; dysferlin, limb girdle muscular dystrophy 2B (autosomal recessive)
hsa:8618	CADPS; Ca++-dependent secretion activator
hsa:23025	UNC13A; unc-13 homolog A (C. elegans)
hsa:63971	KIF13A; kinesin family member 13A ; K10392 kinesin family member 1/13/14
hsa:23095	KIF1B, CMT2A, CMT2; kinesin family member 1B ; K10392 kinesin family member 1/13/14
hsa:2975	GTF3C1; general transcription factor IIIC, polypeptide 1, alpha 220kDa
hsa:547	KIF1A, ATSV, C2orf20; kinesin family member 1A ; K10392 kinesin family member 1/13/14

List of proteins predicted to bind to incednine (Cluster 9)

keggID	protein name
hsa:2195	FAT; FAT tumor suppressor homolog 1 (Drosophila)
hsa:54798	DCHS2, CDH27, PCDH23; dachsous 2 (Drosophila)
hsa:26160	IFT172; intraflagellar transport 172 homolog (Chlamydomonas)
hsa:5243	ABCB1, PGY1, MDR1, CLCS; ATP-binding cassette, sub-family B (MDR/TAP), member 1 ; K05658 ATP-binding cassette, subfamily B (MDR/TAP), member 1
hsa:16	AARS; alanyl-tRNA synthetase (EC:6.1.1.7); K01872 alanyl-tRNA synthetase [EC:6.1.1.7]
hsa:344875	LOC344875; similar to mCG140660
hsa:84059	GPR98, USH2C, MASS1; G protein-coupled receptor 98
hsa:2196	FAT2; FAT tumor suppressor homolog 2 (Drosophila)
hsa:9742	IFT140, WDTC2; intraflagellar transport 140 homolog (Chlamydomonas)
hsa:158326	FREM1, C9orf154; FRAS1 related extracellular matrix 1
hsa:492	ATP2B3; ATPase, Ca++ transporting, plasma membrane 3 (EC:3.6.1.38 3.6.3.8); K05850 Ca2+ transporting ATPase, plasma membrane [EC:3.6.3.8]
hsa:23450	23450 SF3B3; splicing factor 3b, subunit 3, 130kDa
hsa:4842	NOS1, NOS; nitric oxide synthase 1 (neuronal) (EC:1.14.13.39); K00491 nitric-oxide synthase [EC:1.14.13.39]
hsa:31	ACACA, ACAC, ACC; acetyl-Coenzyme A carboxylase alpha (EC:6.4.1.2); K01946 biotin carboxylase [EC:6.3.4.14]; K01961 acetyl-CoA carboxylase [EC:6.4.1.2
hsa:32	ACACB; acetyl-Coenzyme A carboxylase beta (EC:6.4.1.2 6.3.4.14); K01946 biotin carboxylase [EC:6.3.4.14]; K01961 acetyl-CoA carboxylase [EC:6.4.1.2

List of proteins predicted to bind to incednine (Cluster 10)

keggID	protein name
hsa:84163	GTF2IRD2; GTF2I repeat domain containing 2
hsa:389524	GTF2IRD2B; GTF2I repeat domain containing 2B

List of proteins predicted to bind to incednine (Cluster 11)

keggID	protein name
hsa:653489	RGPD3; RANBP2-like and GRIP domain containing 3
hsa:729540	RGPD6; RANBP2-like and GRIP domain containing 6
hsa:84220	RGPD5; RANBP2-like and GRIP domain containing 5
hsa:727851	RGPD8, RANBP2L1; RANBP2-like and GRIP domain containing 8
