# Supplementary figures and images for: Comprehensive predictions of target proteins based on protein-chemical interaction using virtual screening and experimental verifications
Source: BMC Chem Biol. 2012 Apr 5;12:2. doi: 10.1186/1472-6769-12-2 (PMC3471015; doi:10.1186/1472-6769-12-2)

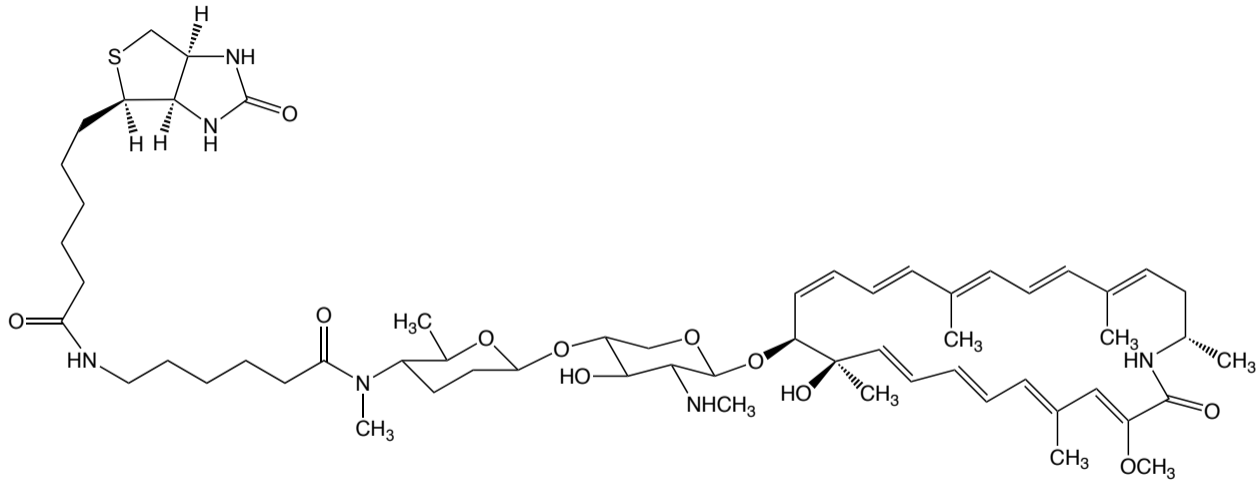

Supplement: Additional file 3 — A stucture of biotinylated incednine. [file 1472-6769-12-2-S3.pdf]
