# Supplementary material for: Comprehensive predictions of target proteins based on protein-chemical interaction using virtual screening and experimental verifications
Source: BMC Chem Biol. 2012 Apr 5;12:2. doi: 10.1186/1472-6769-12-2 (PMC3471015; doi:10.1186/1472-6769-12-2)

Additional file 4 Preparation of Flag-tagged Incednine

Selective amidation of incednine (**1**) of 6-azidohexanoic acid (**2**) using EDCI/DMAP in THF provided azide **3**. Copper-catalyed 1,3-dipolar cycloaddition of **3** with acetylene-containing flag tag **4** ($CuSO_4$/sodium ascorbate/phosphate buffer) was performed at room temperature to afford triazole **5** [46,47].

EDCI = 3-ethyl-1-[3-(dimethylamino)propyl]carbodiimide
DMAP = 4-(dimethylamino)pyridine

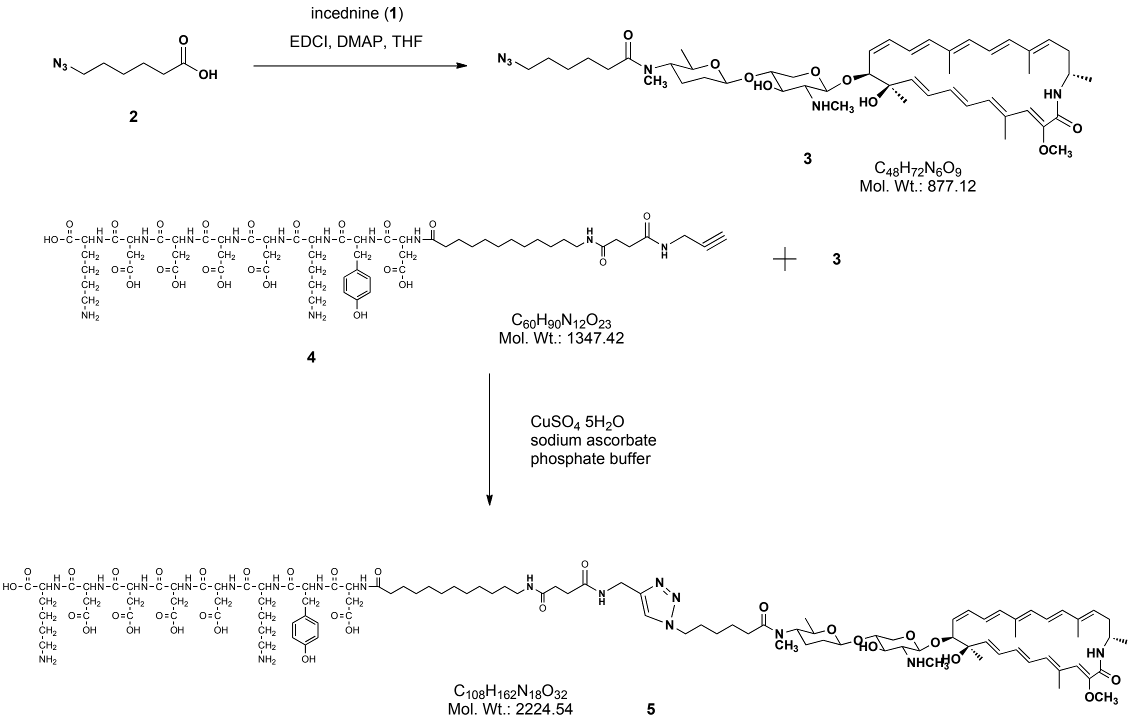

Supplement: Additional file 4 — Preparation of Flag-tagged Incednine[46,47]. [file 1472-6769-12-2-S4.pdf]
